# Association of breast milk gamma-linolenic acid with infant anthropometric outcomes in urban, low-income Bangladeshi families: a prospective, birth cohort study

**DOI:** 10.1038/s41430-019-0498-6

**Published:** 2019-09-09

**Authors:** Josyf C. Mychaleckyj, Dadong Zhang, Uma Nayak, E. Ross Colgate, Marya Carmolli, Dorothy Dickson, Tahmeed Ahmed, Masud Alam, Beth D. Kirkpatrick, Rashidul Haque, William A. Petri

**Affiliations:** 1 Department of Public Health Sciences, University of Virginia, Charlottesville, VA 22908 USA; 2 Center for Public Health Genomics, University of Virginia, Charlottesville, VA 22908 USA; 3 Department of Microbiology and Molecular Genetics, Vaccine Testing Center, University of Vermont Larner College of Medicine, Burlington, VT USA; 4 International Center for Diarrhoeal Disease Research, Dhaka, Bangladesh; 5 Division of Infectious Diseases and International Health, Department of Medicine, Charlottesville, VA 22908 USA; 6 Department of Pathology, University of Virginia, Charlottesville, VA 22908 USA

**Keywords:** Risk factors, Epidemiology

## Abstract

**Background/Objectives:**

Infant linear-growth faltering remains a major public health issue in low- and middle-income countries and suboptimal breast milk composition may be a local, population-specific risk factor. The relationship between early post-natal breast milk fatty acid (FA) composition and infant growth at 1 and 2 years of age was investigated prospectively in 563 families in Dhaka, Bangladesh.

**Subjects/Methods:**

A maternal breast milk sample drawn before infant age 6 weeks was analyzed for percentage composition of 26 FAs, and infant length for age *Z* score (LAZ) was measured longitudinally to infant age 2 years. Individual FAs were tested as predictors of the infant growth outcomes.

**Results:**

Of 26 tested FAs, %gamma-linolenic acid (%GLA) was mostly significantly associated with increase in LAZ from 6 to 52 weeks (ΔLAZ(52−6w)), and also to 104 weeks. The association was consistent over all breast milk stages with estimated effect size of +0.05 ΔLAZ(52−6w) per 20% change in %GLA (*p* value = 3 × 10^−6^), and stronger for ΔLAZ(104−6w) at +0.06 (*p* value = 8 × 10^−7^), explaining 1% of the outcome variance. Infant serum zinc measurements at 6 and 18 weeks of age were included in adjusted analyses, suggesting at least partial independence of infant zinc levels. The association was strongest in 417/563 (74.1%) families with %GLA <0.2%. Breast milk arachidonic acid fraction was within normal range with weaker evidence of association in early breast milk stages.

**Conclusions:**

This study found that %GLA in breast milk was independently associated with infant linear growth, albeit with small effect size, in a predominantly slum-dwelling, low-income, Bangladeshi cohort.

## Introduction

Breast milk from well-nourished mothers supplies complete nutrition for a neonatal infant, with a composition that adjusts to meet the changing metabolic and nutrient needs of the infant [1]. The critical importance of exclusive breastfeeding to support early infant development is well-established and is especially effective in low- and middle-income countries where delay in complementary feeding can reduce the nutritional impact of food insecurity on the infant and direct ingestion of pathogens, while providing passive protection as the nascent infant immune system matures [2, 3]. Although systematic meta-analyses have largely failed to find a significant effect for breastfeeding interventions on post-natal infant anthropometric outcomes [4], the estimated effects are highly heterogeneous, with country income and intervention setting contributing the greatest proportion of heterogeneity [5]. The positive infant outcomes derived from exclusive breastfeeding rest on the assumption that expressed milk contains the requisite balance of macro- and micronutrients to sustain the infant at each stage of infant development. Long chain polyunsaturated fatty acids (LCPUFAs) are one class of micronutrient for which there is a strong biological basis for a key role in infant physical growth and cognitive development [6], but maternal or infant supplementation with LCPUFAs has resulted in little significant improvement in post-natal anthropometry outcomes through 1 or 2 years of life [7–9]. This suggests that if deficiencies exist that affect infant growth, they are specific and local.

We previously recruited and followed a cohort of low-income, mother−infant families in Bangladesh, many living in slums, to study vaccine response and infant growth as components of the PROVIDE Study (Performance of Rotavirus and Oral Poliovirus Vaccines in Developing Countries) [10]. The mothers in the cohorts were given health education, primary care, and encouraged to exclusively breastfeed according to recommended guidelines, but with the high prevalence of micronutrient deficiency in women of reproductive age in Bangladesh [11–13], we were concerned that the mothers may have fed breast milk deficient in micronutrients essential for infant growth. Focusing on the long chain saturated and unsaturated fatty acid (FA) micronutrient composition, we hypothesized that deficiencies in the FA composition of breast milk would be associated with impaired infant linear or ponderal growth [6]. We employed an agnostic analytical approach to look beyond the usual focus on a few key FAs to consider all 26 FAs as equally plausible candidate growth-promoting micronutrients, including intermediate metabolites in the omega-6 and omega-3 FA pathways.

## Methods

### Study population and design

The PROVIDE study Bangladesh cohort has been reported previously in papers describing the study design [10] and clinical trial outcomes [14, 15]. Further details of Methods are available in Supplementary Materials. Briefly, 700 eligible mothers with their newborns, living in predominantly low socio-economic status households, many in slum conditions in the study recruitment area in Dhaka, Bangladesh, were consented within 7 days post-delivery into the PROVIDE study. The study inclusion/exclusion criteria are listed in Supplementary Table S1. This study was conducted according to the guidelines laid down in the Declaration of Helsinki and all procedures involving human subjects/patients were approved by the Ethical Review Committee for human subjects protection (protocol # PR-10060), and Research Review committee for scientific merit at the International Centre for Diarrhoeal Diseases research, Bangladesh (icddr,b); and by the Institutional Review Boards at the University of Virginia (HSR 15377, sponsor protocol # CHRMS:M11-112), and University of Vermont (sponsor protocol # CHRMS:M11-112). Written informed consent was obtained from all subjects.

### Procedures

Infant anthropometry was measured up to 2 years of age, at enrollment and 14 additional visits at weeks 6, 10, 12, 14, 17, 18, 24, 39, 40, 52, 53, 78, 91, and 104. Trained field research staff measured infant weight to the nearest 10 g and infant recumbent length to the nearest 0.1 cm in duplicate. If these were not within 10 g for weight and 0.1 cm for length, a third measurement was taken and the average of two measurements within this tolerance was recorded and used to calculate WHO standardized *Z*-scores, length-for-age (LAZ), weight-for-age (WAZ), and weight-for-height (WHZ). Infant gestational age was estimated for a subset of the infants to distinguish fetal growth restriction from prematurity using the Dubowitz−Ballard assessment scale [16, 17]. Breast milk FA assay procedures have been described previously and are reiterated in Supplementary Methods [18–20]. Individual breast milk fatty acids were expressed as %wt/wt of total identified FA; the FA profile of each specimen contained 26 individual FAs, listed in Supplementary Table S2. Infant serum zinc (μg/l) was measured using flame atomic absorption spectrophotometry. Serum zinc deficiency was defined as <640 μg/l [21].

### Outcomes

The primary outcome was the change in infant length-for-age *Z* score (LAZ) from 6 to 52 weeks of age. The secondary outcomes were the change in LAZ from 6 to 104 weeks of age, and corresponding change in WAZ over the same time intervals.

### Statistical analyses

Descriptive statistics of continuous variables were expressed as means ± standard deviations, and dichotomous variables as proportions. The FA measures were bounded in the composition range [0, 100%], many were skewed, and the Akaike Information Criterion (AIC) confirmed that the log-transformed variable resulted in a significantly better fit; therefore, all predictor FA composition percentage variables were log-transformed in the statistical models. %AA and %DHA were preselected candidates but the other 24 FAs were given equal prior weight as possible predictors of the anthropometry outcomes. A two-stage analysis was used: (1) predictor FA variable selection; (2) extensive multiple regression testing. In stage one, the Least Absolute Shrinkage and Selection Operator (LASSO) variable selection method was used to select other non-preselected significantly associated FAs at each step maximizing a penalized likelihood that shrinks nonselected variable coefficients [22, 23]. The advantage of this method is that a significance test can be applied to each stepwise selected variable that adjusts the degrees of freedom to account for multiple testing of candidate variables. FA variables significant at 0.05 in the LASSO covariance test were carried forward to the second stage of analysis together with preselected %AA and %DHA. Detailed discussion of power for FA detection is described in Supplementary Methods and Table S3. In stage two we used multiple linear regression to estimate the effects of the selected FA composition variables after adjustment. The minimally adjusted regression models (‘Minimal’ model) contained the following preselected variables for adjustment: breast milk arachidonic and docosahexaenoic acid, log(%AA) and log(%DHA); infant serum zinc at week 6 and week 18; infant age at breast milk sample; infant age at 6 and 18 zinc serum samples; and infant age at week 6 and week 52 (or 104) anthropometry visits. Gestational age was tested as an adjustment in the Minimal model, but where it did not significantly alter an association, the Minimal model results were preferred for increased sample size; estimated gestational age was only available for a subset of the infants. Stratified breast milk stage analyses used the intervals: colostrum, <6 days; transitional, 6–15 days; mature, >15 days. Effect sizes and confidence intervals for the log-transformed %FA predictor variables were presented as untransformed to preserve additivity, or back-transformed to a multiplicative effect size of change in outcome per 20% change in the %FA composition, depending on circumstances. All statistical analyses used R version 3.4. The LASSO analysis used the lars package (https://cran.r-project.org/package=lars).

## Results

### Clinical characteristics

**Fig. 1:**
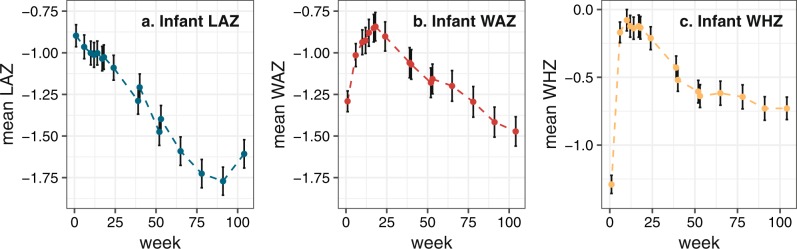
Plots of the longitudinal change in mean LAZ (**a**), WAZ (**b**), and WHZ (**c**) for infants in the PROVIDE study Bangladeshi cohort. Vertical bars indicate estimated 95% confidence intervals of the means. Anthropometric data were available to week 104 by protocol design. LAZ length for age *Z* score, WAZ weight for age *Z* score, WHZ weight for height *Z* score. The *y*-axis ranges for LAZ and WAZ plots are the same but WHZ is dissimilar

Of 700 families, 563 (80%) provided a breast milk sample that resulted in FA data and were retained in the study with complete infant serum zinc and weeks 6 and 52 anthropometry data, the minimum necessary for primary outcome analyses (CONSORT diagram, Supplementary Fig. S1). The clinical characteristics of mothers and infants are shown in Table 1, at enrollment, study weeks 6, 18, 52, and 104. The infants were a mean of 5 days old (range 0–7 days), predominantly born outside the home (74%) into families subsisting on a mean of approx 140USD per month, and were almost all breastfed at birth (95%). At 6 weeks, the infants were moderately-to-severely linear growth-impaired (mean WHO length-for-age *Z* score, LAZ = −0.96; 12% stunted with LAZ < −2) and 22% were serum zinc deficient, falling to 15% at week 18. Figure 1 shows the longitudinal change in LAZ, weight-for-age and weight-for-height *Z* scores (WAZ, WHZ), at site-specific study visits. The mean LAZ dropped almost inexorably starting immediately after birth, while the WAZ rose to a maximum at approximately week 18, 1 week after the mean end of exclusive breastfeeding at 17 weeks (Table 1), then dropped. These trends were also reflected in the proportion (28%) of stunted infants at 52 weeks, rising to 33% at 104 weeks.

**Table 1:** Clinical characteristics of the PROVIDE infant and mother participants

Characteristic	Value (SD or %)			Value (SD or %)
**Infant enrollment visit (0−1 week)**	*n* = 700	**Mother enrollment visit (0−1week)**^a^		*n* = 700
Enrollment age, days	5 (1.7)	Age, year		24.7 (4.6)
Sex (Male), *n* (%)	368 (52.8)	Postpartum BMI, kg/m^2^		21.8 (3.7)
Birth order	1.9 (0.8)	Height, cm		150.3 (5.5)
Nuclear family only in dwelling, *n* (%)	414 (59.1)	First pregnancy, *n* (%)		213 (30.4)
Infant gestational age, weeks^b^	37.6 (1.4)	Education		
Infant gestational age ≤ 36 weeks, *n* (%)^b^	123 (32.3)	None, *n* (%)		202 (28.9)
Breastfed at birth, *n* (%)	662 (94.6)	Primary, *n* (%)		263 (37.8)
Home birth, *n* (%)	181 (25.9)	Secondary or higher, *n* (%)		235 (33.6)
Monthly household expenditure, 1000 s Taka^c^	11.5 (7.2)	Occupation homemaker (%), *n* (%)		601 (85.9)
		Infant age at breast milk sample, days^d^		10.4 (6.3)
**Infant visit**	**6 weeks (*n*** = 652)	**18 weeks (*n*** = 590)	**52 weeks (*n*** = 605)	**104 weeks (*n*** = 578)
Age, days	44.7 (2.8)	134.0 (5.2)	369.0 (6.4)	737.2 (6.5)
Length, cm	54.1 (1.9)	—	71.4 (2.7)	81.7 (3.4)
Weight, kg	4.27 (0.54)	—	8.2 (1.1)	10.1 (1.4)
LAZ	−0.96 (0.93)	—	−1.48 (1.02)	−1.61 (1.04)
Stunted LAZ < −2, *n* (%)	81 (12.4)	—	167 (27.6)	191 (33.0)
WAZ	−1.01 (0.90)	—	−1.18 (1.11)	−1.47 (1.08)
Underweight WAZ < −2, *n* (%)	85 (13.0)	—	133 (22.0)	174 (30.1)
WHZ	−0.17 (0.98)	—	−0.61 (1.04)	−0.73 (1.01)
Wasted WHZ < −2, *n* (%)	14 (13.0)	—	45 (7.4)	47 (8.1)
ΔLAZ (52/104 weeks−6 weeks)	—	—	−0.51 (0.85)	−0.64 (0.95)
ΔWAZ (52/104 weeks−6 weeks)	—	—	−0.18 (0.90)	−0.46 (0.99)
ΔWHZ (52/104 weeks−6 weeks)	—	—	−0.46 (1.14)	−0.57 (1.18)
Serum zinc, μg/l	726 (111)	772 (147)	—	—
Zinc deficient (serum zinc < 640 μg/l), *n* (%)	139 (22.0)	91 (15.4)	—	—
Serum CRP, mg/l	1.0 (3.7)	3.0 (7.7)	—	—

Continuous data summaries are mean (SD), or categorical *n* (%) where indicated; — signifies measures not applicable for this analysis

*BMI* body mass index, *LAZ* length for age *Z* score, *WAZ* weight for age *Z* score

^a^Maternal characteristics were collected at infant enrollment (age, postpartum BMI)

^b^Infant gestational age was measured on a subset of 333 infants in Bangladesh to distinguish fetal growth restriction from prematurity using the Dubowitz-Ballard assessment scale

^c^During the study period 1 USD = approximately 80 Bangladesh Taka

^d^Range of infant age at breast milk sample was 3–43 days

### Breast milk composition

The breast milk specimens were collected during a range of infant ages 3–43 days (mean 10.6), hence 72/563 (12.8%) were classified as colostrum milk (≤5 days), 409/563 (72.6%) as transitional (6–15 days), and 82/563 (14.6%) as mature (>15 days). Supplementary Table S2 shows the complete set of 26 FAs assayed, their mean breast milk percentage in all mothers with data (*n* = 683), and in the families with complete outcome and covariate data (*n* = 563). Of note, our total sample mean (SD) values for %AA and %DHA were 0.53 (0.14) and 0.39 (0.13) respectively.

### Selection of fatty acids associated with infant growth

Running the LASSO procedure for association with the primary outcome weeks (ΔLAZ 52–6 weeks) for all 26 FAs (including AA and DHA) resulted in percentage of gamma-linolenic acid (%GLA) being selected at step one with overwhelming association statistical significance despite the in-built correction for multiple FA testing (*p* value = 0.00001) (Table 2). The second and subsequent stepwise selected FAs were not significant after correction. Surprisingly, AA was only selected as the fifth-best sequential explanatory FA (*p* value = 0.8). These results were also true for ΔLAZ (104−6 weeks). The same procedure applied to ΔWAZ (52–6 weeks) and ΔWAZ (104–6 weeks) outcomes did not select any FAs at a stepwise *p* value < 0.05.

**Table 2:** Results from stepwise selection of breast milk-measured fatty acid composition associated with linear growth faltering outcomes using a penalized selection procedure

Outcome	Selection step	Selected fatty acid^a^	*p* value for stepwise selected fatty acid
Primary			
ΔLAZ (52–6 weeks)	1	GLA	0.00001
	2	LA	0.94
	3	EDA	0.6
Secondary			
ΔLAZ (104−6 weeks)	1	GLA	0.0011
	2	LA	0.59
	3	PLA	0.54
ΔWAZ (52−6 weeks)	1	GLA	0.15
	2	LA	0.42
	3	PAL	0.93
ΔWAZ (104−6 weeks)	1	LA	0.09
	2	AA	0.25
	3	DGLA	0.88

A stepwise *p* value of 0.05 was considered significant. Fatty acid variable selection ceased after the first nonsignificant selected fatty acid

*LAZ* length for age *Z* score

^a^Fatty acid abbreviations: *AA* arachidonic acid, *DGLA* dihomo-gamma-linolenic acid, *DHA* docosahexaenoic acid, *EDA* eicosadienoic acid, *GLA* gamma-linolenic acid, *LA* linoleic acid, *PAL* palmitic acid, *PLA* palmitolaidic acid

### Association of breast milk %gamma-linoleic acid with infant growth

**Fig. 2:**
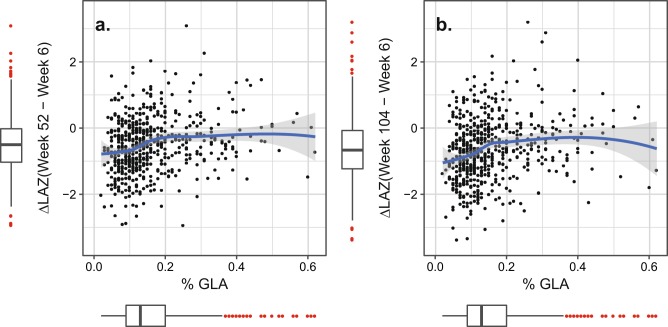
Plots of the unadjusted primary and secondary outcomes, ΔLAZ (week 52–week 6) and ΔLAZ (week 104–week 6) against the percentage of gamma-linolenic acid (%GLA) in breast milk in the PROVIDE study Bangladeshi families. The blue line is the nonlinear local mean at each value of %GLA fitted by loess algorithm, and the gray envelope is the 95% confidence envelope. Red points in surrounding box plots indicate points outside the range [median – 1.5 × interquartile range, median + 1.5 × interquartile range]. LAZ length for age *Z* score. To magnify the change of interest, the *y*-axis range of the plots omits one extreme outlier infant point at ΔLAZ(week 52−week 6) = −5.19, %GLA = 0.04; and the same infant ΔLAZ(week 104 − week 6) = −4.49, %GLA = 0.04

Multiple linear regression analyses using the Minimal model, including preselected AA and DHA as possible true associated trophic FAs, confirmed that log(%GLA) was the most significant FA variable (Table 3, *p* value = 3 × 10^−6^, *n* = 563). The effect size was not different between sexes (interaction test *p* value = 0.38) and remained significant after adding gestational age to the Minimal model adjustments in a subset of the families (*p* value = 7 × 10^−4^, *n* = 318). The transformed adjusted effect size of breast milk %GLA on mean ΔLAZ(52–6 weeks) under the Minimal model was +0.05 ΔLAZ units per 20% increase in %GLA 95%CI[+0.03, +0.07] (Table 3). The population mean level of %GLA in breast milk was 0.16% (SD = 0.10%) (Supplementary Table S2). The %GLA association did not change with continuous sample time point (interaction test of age of infant at breast milk sample and log(%GLA), *p* value = 0.33). To further test for an association with %GLA resulting from an underlying zinc deficiency in the infants, we reran the models having removed any infant apparently zinc deficient (<640μg/l) at 6 or 18 weeks of age (Table 1), retaining the zinc levels at 6 and 18 weeks as adjustments in the model. Again log(%GLA) retained significance as an independent predictor of ΔLAZ(52–6 weeks) with effect size +0.04 [+0.01,+0.06], *p* value = 0.0025. We performed a similar test for maternal zinc deficiency, using breast milk %LA/%DGLA ratio as a proxy [24]. Inclusion of this ratio in the Minimal model still did not eliminate the %GLA association for ΔLAZ(52–6 weeks) (*p* value = 0.0063). Plots of the primary outcome versus %GLA revealed the families where reduced %GLA was associated with the largest length growth deficit (Fig. 2). Mean ΔLAZ (52−6 weeks) increased with increasing %GLA at low concentrations of %GLA <0.2%, with 0.2% as a threshold, above which additional increase in %GLA was not associated with further increase in the outcome. The trends and conclusions remained unchanged after adjusting the primary outcome (Supplementary Fig. S2). There were 417/563 (74%) of mothers below a %GLA threshold of 0.2%. Interestingly, the association was stronger for the secondary outcome, ΔLAZ (104−6 weeks) (Minimal effect size +0.06 [+0.04, +0.09], *p* value = 8 × 10^−7^), but less significant for the ΔWAZ outcomes (Table 3).

**Table 3:** Results for the association of percent gamma-linolenic acid in breast milk with linear growth faltering outcomes in the PROVIDE Study

Outcome	*n*	Model^a^	Effect size per 20% change in %GLA [95%CI]^b^	*p* value^c^
Primary				
ΔLAZ (52–6 weeks)	563	Minimal	0.05 [0.03, 0.07]	3 × 10^−6^
	317	Minimal + GA	0.05 [0.02, 0.08]	7 × 10^−4^
Secondary				
ΔLAZ (104–6 weeks)	538	Minimal	0.06 [0.04, 0.09]	8 × 10^−7^
	298	Minimal + GA	0.05 [0.02, 0.09]	0.003
ΔWAZ (52–6 weeks)	563	Minimal	0.04 [0.02, 0.07]	1 × 10^−4^
	317	Minimal + GA	0.05 [0.02, 0.09]	0.002
ΔWAZ (104–6 weeks)	538	Minimal	0.03 [0.005, 0.06]	0.02
	298	Minimal + GA	0.05 [0.02, 0.09]	0.007

The primary outcome was the change in LAZ from 6 weeks to 52 weeks

*LAZ* length for age *Z* score, *WAZ* weight for age *Z* score

^a^Minimal model adjustments: sex, log(%AA), log(%DHA), infant serum zinc (week 6), infant serum zinc (week 18), infant age at anthropometry and zinc measures, infant age at breast milk sample Minimal + GA: added gestational age to the Minimal model

^b^%GLA in breast milk was tested as log(%GLA). Effect sizes and 95% CI were converted to a standard effect size of the change in outcome ΔLAZ or ΔWAZ for a 20% increase in %GLA. For comparison, one SD increase in %GLA at the estimated mean %GLA (0.16%) would be a 62.5% increase in breast milk %GLA

^c^The *p* value tests the significance of the association of log(%GLA) with the anthropometry outcome

### No independent association of breast milk %AA and %DHA with infant growth

In the same Minimal model for %GLA, we saw no independent association of the preselected %DHA (*p* value = 0.69) (Supplementary Table S4), but we did see a nominal association with %AA (*p* value = 0.019). However, when we added the estimated infant gestational age at birth to the Minimal model adjustments for the subset of the families with gestational age measured, the association was attenuated to nonsignificance (Supplementary Table S4, *p* value = 0.071, *n* = 318).

### Association of %GLA, %AA, and %DHA with infant growth by breast milk stage

**Fig. 3:**
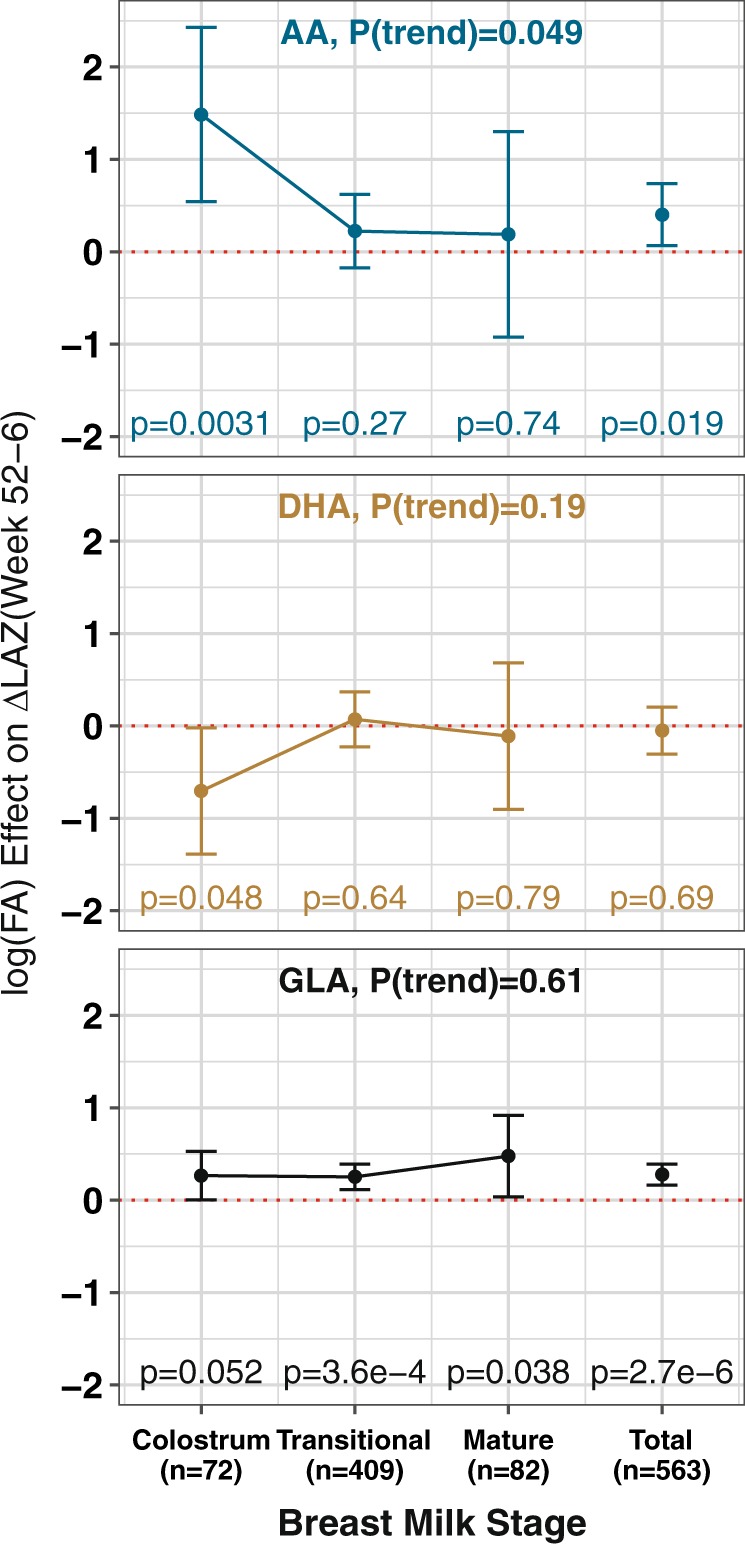
Plots of the association effect size (beta) for breast milk %AA, %DHA, and %GLA for the primary outcome ΔLAZ(week 52−week 6), stratified by breast milk stage and sample size (*n*) for that stage. All three log(%FA) were included in each model. Effect sizes are in log(%FA) units. Error bars indicate the 95%CI range. Individual stage and FA *p* values are shown for each beta. *P*(trend) is the *p* value for a test of trend in the effect sizes from colostrum to mature, *P*(trend) < 0.05 indicates a significant change in effect size by stage

We stratified tests of association under the Minimal model by breast milk stage (Fig. 3 and Supplementary Table S4). The primary association with %GLA was present in all three stages and largely unchanged in direction or magnitude (*p* value trend = 0.61), accounting for the very significant *p* value for the overall test, including adjustment for continuous age of infant at sample. %AA was apparently more strongly and significantly positively associated in the colostrum stage than %GLA or %DHA (*p* value = 0.0031) but was attenuated in transitional and mature stages (*p* value trend = 0.049); hence, the apparent overall association with %AA was solely driven by the colostrum stage (*n* = 72). %DHA was more weakly and less significantly associated in colostrum stage (*p* value = 0.048) and like %AA, attenuated to insignificance in the sample-size-reduced gestational age-adjusted model.

## Discussion

The percentage of gamma-linolenic acid (%GLA) in breast milk sampled up to 6 weeks postpartum was prospectively positively associated with change in infant LAZ from 6 to 52 and to 104 weeks of age in low-income, predominantly slum-dwelling, families in Mirpur, Dhaka, Bangladesh. The association was present at all stages of lactation and was strongest in the 74% of families where the %GLA was below 0.2%. The association remained significant after adjusting for possible confounders including infant serum zinc concentration at 6 and 18 weeks of age, and %AA and %DHA suggesting that the association of %GLA is partially or wholly independent of infant serum zinc status and is not a phantom association for %AA or %DHA compositional factors. An apparently stronger association of the infant change in LAZ outcome with %AA limited to the colostrum stage was attenuated to nonsignificance after adjustment with gestational age, and may have represented confounding, but could also have diminished due to reduced sample size. Pervasive secondary associations with other FA members of the omega-6 pathway in the analyses, seen in the nonsignificant, nonselected second FAs in the LASSO and regression models, suggests that the individual %GLA association is not an outlier and that there is some relative deficiency in the omega-6 pathway in this population. The stronger evidence for association of omega-6 breast milk PUFA variation suggests a greater biological role in infant linear growth than omega-3 PUFAs, including DHA.

Our finding that GLA was the most significantly associated with infant linear growth was unexpected although GLA supplementation can increase infant growth [25]. The positive association with ΔLAZ was small in magnitude, with an estimated mean change of +0.05 per 20% increase in %GLA for ΔLAZ (52–6 weeks) and slightly larger for ΔLAZ (104–6weeks), +0.06. The percentage of variance in the outcome explained by %GLA was about 1% for both ΔLAZ outcomes.

A plausible a priori explanation for the association was that the %GLA content was a proxy for a true underlying deficiency in the %AA breast milk content. Our models all included %AA or %DHA as covariate test variables; hence, the residual %GLA association was independent of their effects and they did not attenuate the association of %GLA to insignificance. Comparing the mean %AA and %DHA in our breast milk samples to published global values [26], our total sample mean(SD) values for %AA and %DHA were 0.53(0.14) and 0.39(0.13) compared to global mean 0.47(0.13) and 0.32(0.22) respectively (84 studies, *n* = 2474). For the 27/84 studies with breast milk collected up to 2 months postpartum, our mean values of %AA and %DHA ranked above the median value, with multiple high-income country studies showing lower mean %AA and %DHA than the PROVIDE families. Hence our means did not appear especially low. Mean %AA was higher in colostrum than %DHA but %GLA was lower than both %AA and %DHA in colostrum, so greater %FA did not consistently result in more significant association with infant growth overall or in a stratified breast milk stage.

One possible explanation for these results is an underlying pathology of zinc deficiency in a subset of the mothers which could have led to impaired conversion of essential LA to GLA by reduced Δ6-desaturase efficacy [27, 28]. We did not measure maternal breast milk or serum zinc levels, but 20% of the infants were zinc deficient at 6 weeks, and 15% at 18 weeks, suggesting possible maternal zinc deficiency during the largely exclusive breastfeeding early neonatal period. Since 99% of AA in breast milk is mobilized from stored maternal depots [29, 30], dysregulation of this enzymatic step would be expected to affect both GLA and AA, although secondary effects of zinc deficiency on FA elongation and Δ5-desaturation could also affect subsequent enzymatic conversion of GLA to AA. Inclusion of the %LA/%DGLA proxy ratio [24] in our models did not eliminate the %GLA effect. Maternal zinc deficiency remains a possibility, but does not seem to fully explain the association.

Since endogenous omega-6 and omega-3 pathways are active in term and preterm neonatal infants [31, 32], the zinc deficiency could extend to infant metabolism. The inclusion of infant serum zinc concentrations at 6 or 18 weeks of age did not eliminate the association with %GLA, as would be expected if low zinc were the only causative factor. Another possibility is that the association is a marker of the effects of future zinc deficiency, after the infant has transitioned to complementary feeding or has weaned (mean 16 weeks). The nutritional deficiency is likely family-specific, but has less effect in the breastfeeding infant because of the milk supply and prioritization of the mother’s reserves to supply the infant. Due to lack of data on plasma zinc levels in the post-breastfeeding infants, we cannot test this hypothesis.

If GLA in breast milk is functionally associated with infant linear growth, there are at least two possible mechanisms for such an effect. If AA is the only true trophic FA metabolite factor in the omega-6 pathway affecting infant length, then GLA and perhaps other upstream components from AA may represent reservoirs that are diverted to AA synthesis in the infant under conditions of extreme undernourishment. GLA is the most distal upstream omega-6 after the first rate-limited Δ6-desaturase step, and hence could be the omega-6 PUFA of last metabolic resort for future conversion to AA in times of nutritional demand, when the mother is unable to deliver sufficient total volume of preformed growth metabolites in her milk. GLA is only a small fractional component of breast milk, although total infant FA intake depends on total milk intake. Alternatively, GLA may be an independent growth factor, in addition to its role as a precursor for downstream elongation and desaturation. GLA and DGLA are also known to have secondary biological activity [33]. DGLA can be converted into anti-inflammatory eicosanoids with clinical efficacy in treating chronic disease-related processes of inflammation [34]. Supplementation with dietary GLA can directly elevate DGLA, helping to counter the proinflammatory overproduction of eicosanoids from AA [35]. The association of GLA with infant linear growth may not have been previously recognized because prior studies with breast milk and anthropometry data were either underpowered; did not employ a hypothesis-neutral approach for breast milk FA predictors and only focused on AA and DHA; or did not test the association in a population where this specific type of malnutrition is prevalent.

Our study has limitations. We assayed wt/wt percentage of each FA in a single breast milk sample and did not measure the total intake of the FA by the infant. The breast milk composition could be a marker of deficiency both pre- and post-weaning. This was an observational study with its attendant limitations of causative inference, but we ameliorated this by performing modeling to reduce confounding bias. We cannot definitively rule out other untested confounders, but our analyses suggested that %GLA was the most significant risk factor, albeit not the FA of largest mean effect size. Between 15 and 20% of the infants were zinc deficient, and zinc deficiency is likely also contributing to growth deficiency but despite all of our efforts to explain the %GLA association by zinc levels in the infants, %GLA remained as an independent associated linear growth factor. Our adjustments for gestational age resulted in a very reduced sample size.

In summary, this study found that %GLA in breast milk is independently associated with early infant linear growth up 2 years in a low-income, predominantly slum-dwelling, Bangladeshi cohort through all stages of breast milk composition, although the effect size is small. We hypothesize that there is a relative omega-6 deficiency in this population, from an imbalance in breast milk composition or impaired post-breastfeeding infant metabolism, that affects linear growth. Even after transition to complementary feeding or weaning, the infant is unable to catch up the growth deficit through 2 years of age. Replication is needed to confirm this result and may require specific circumstances of omega-6 deficiencies, a situation likely uncommon in higher income countries.

## Supplementary information

Supplemental Materials
