# Supplementary material for: Association of breast milk gamma-linolenic acid with infant anthropometric outcomes in urban, low-income Bangladeshi families: a prospective, birth cohort study
Source: Eur J Clin Nutr. 2019 Sep 9;74(5):698–707. doi: 10.1038/s41430-019-0498-6 (PMC7214250; doi:10.1038/s41430-019-0498-6)
Supplement: Supplementary file 1 — Supplemental Materials [file 41430_2019_498_MOESM1_ESM.docx]

**Association of breast milk gamma-linolenic acid with infant anthropometric outcomes in urban, low-income Bangladeshi families: a prospective, birth cohort study.**

**Mychaleckyj JC et al.**

**Supplementary Methods**

***1. Study Population, Design, Eligibility***

Seven hundred (700) eligible low-income neonatal infant–mother families were enrolled in the slum areas of the Mirpur Thana, Dhaka, Bangladesh between May 2011 and November 2012. The cohort sample sizes were designed to give necessary statistical power for infant poliovirus and rotavirus vaccine interventional clinical trials conducted simultaneously in the cohorts. The infant poliovirus and rotavirus vaccine trials ran contemporaneously with the longitudinal infant growth and development study, and the mother was not subject to vaccine intervention. The eligibility criteria are listed in Supplemental Table S1. Eligibility for enrollment in Bangladesh required delivery of a live infant of maximum age 7 days, absent frank congenital abnormalities, birth defects, or irregular stool frequency or consistency. The mother was required to have no immediate plans to leave the study recruitment area, non-participation in other interventional studies, and willingness to accede to all study protocol data collection and specimen sampling. Given these criteria, enrollment was random.

***2. Procedures***

Maternal and socioeconomic factors associated with the major fatty acid fractions in breast milk composition in this cohort has been reported, but did not include the detailed fatty acids (FAs) [Nayak et al Matern Child Nutr 2017] nor infant data. Infant anthropometry was measured up to two years of age, at enrollment, and 15 additional visits at weeks 6, 10, 12, 14, 17, 18, 24, 39, 40, 52, 53, 78, 91, and 104. Field research staff measured infant weight to the nearest 10g using a well-calibrated Seca 354 Digital Baby Scale, and infant recumbent length to the nearest 0.1cm using a Seca 416 Infantometer. The measurements were taken in duplicate by trained personnel in presence of the physicians in the study clinic. The training included proficiency in anthropometry measurement with a maximum of 5% deviation from a standard measurement taken on a volunteer infant by a senior field research staff member with extensive experience in anthropometry. Any field research assistant who did not meet the maximum 5% deviation from the standard measurement received further training and did not perform study measurements until she could meet the target reliably. Training and testing for anthropometry was repeated and documented annually for every field research assistant.

If two measurements were not within nearest 10g for weight and 0.1cm for length, a third measurement was taken and the average of two acceptable measurements was utilized to calculate the WHO standardized Z-scores, length-for-age (LAZ), weight-for-age (WAZ), and weight-for-height (WHZ). We adopted the WHO standard Z-score thresholds of < -2 to define stunting (LAZ < -2) and underweight (WAZ < -2) . Breast milk specimens were collected by trained field research assistants during surveillance home visits. Mothers were guided to manually express approximately 5mL breast milk from the pre-cleansed nipple of their choice of breast into a pre-labeled falcon tube without restriction to fore or hind milk.

A single breast milk specimen was scheduled for collection from each mother at 6 weeks postpartum, although the exact collection date varied due to missed and rescheduled study visits. The specimens were transported to laboratories at icddr,b in insulated carriers with cold packs at 4degC. At the laboratories, after preparation of a 50ul dried milk spot and anti-oxidant pre-treatment, the milk spot cards were shipped to OmegaQuant Analytics laboratory, Sioux Falls, South Dakota, for analyses by gas chromatography.

***3. Breast Milk Inter-Assay Coefficient of Variability***

We estimated the inter-assay coefficient of variation for the dried milk spot / gas chromatography protocol from published data [Jackson et al, Int. Breastfeed. J. 2016] on 5 sample replicates run at OmegaQuant labs in the US and Asia. The results are shown in Supplementary Table 2, inter-assay, % coefficient of variation. Only 4/26 FAs had inter-assay %CV>20.0%, and the omega-6 pathway FA %CVs range was from 5.5-15.5%.

***4. Confounders and Explanatory Factors***

The log(%AA) and log(%DHA) were pre-selected based on prior literature to test their primary effects, but also whether any other significant FA was a confounded proxy for an association with one or both of these possibly ‘true’ underlying trophic FAs. Since deficiency of GLA and disturbed ratios of proximal omega-6 FAs have been linked to zinc deficiency, the inclusion of infant zinc measure at week 6 was designed to test this possibility. The week 18 serum zinc measure was also included as potentially associated with the outcome to reduce outcome variance. We considered it very unlikely that week 6 breast milk FAs would be directly associated with week 18 serum zinc as a mediator. Inclusion of confounding or mechanistic variables in the model were expected to reduce or eliminate the statistical significance of any FAs carried forward for hypothesis testing in the linear models and hence greater probability of accepting the null hypothesis of no association with breast milk FAs.

***5. Power and Sample Size Considerations***

The total family sample size was constrained to be 700 minus sporadic missing data and loss to follow-up. We computed our power to detect each of the 26 fatty acids using the LASSO procedure in 563 families after drop-out and sporadic missing data. We simulated linear models of association using the study estimated FA correlation matrix, and mean and variance of the FAs, computed from 683 total mothers with breast milk results. Each FA was individually simulated to be associated with the primary outcome by converting the fixed proportion of variance in the outcome explained by the log(FA) to a non-zero effect size ($\beta_{1})$:

$${\Delta LAZ}_{i}=\beta_{0}+\beta_{1}\log\left( {FA}_{ij} \right)+ e_{i} ; cov\left( {log(FA)}_{ij}, {log(FA)}_{ik} \right)= \Sigma_{jk}$$

where $\Sigma$ is the covariance matrix of the 26 FAs expressed as log(FA). We calculated the power for each FA individually as the proportion of 1000 simulations in which that FA as the first selected by the LASSO procedure and with a p-value< 0.05. The detailed results are shown in Supplementary Table S3. In all cases, LA and DTA FAs had the lowest power for LASSO selection. We had ≥80.0% power to detect any breast milk FA that explained at least 5% of the total primary outcome variance. For 2% variance explained, the power varied from 34.7% to 54.7% and for 1% explained, 11.2% to 22.9%. Post-hoc, we note that the only LASSO-selected FA for the primary outcome achieved a p-value of 0.034 in the fully adjusted ‘causative’ model with a log(FA) effect size of +0.079 change in infant LAZ, suggesting that this is close to the minimal detectable size for this FA. All else being equal, p-value=0.05 in this test would detect a ΔLAZ effect size of +0.073 (7.6% smaller).

**Supplementary Results**

***1. Association of Breast Milk %Gamma-Linoleic Acid with Infant Growth***

To further test for an underlying zinc deficiency in the infants, we reran the models having removed any infant who appeared to be zinc deficient (<640ug/L) at 6 or 18 weeks of age (Table 1), retaining the zinc levels at 6 and 18 weeks as adjustments in the model. Again log(%GLA) retained significance as an independent predictor of ΔLAZ(52–6 weeks) with effect size +0.04 [+0.01, +0.06], p-value=0.0025. We performed a similar test for maternal zinc deficiency, using breast milk %LA/%DGLA ratio as a proxy. This ratio was associated with the infant concentration of zinc measured at 18 weeks of age, but inclusion of this ratio in the Minimal model still did not eliminate the %GLA effect for ΔLAZ(52–6 weeks) (p-value=0.0063).

***2. Association of %GLA, %AA, and %DHA with Infant Growth by Breast Milk Stage***

The association of %AA in colostrum was attenuated to insignificance when gestational age was added to the Minimal model (p-value=0.26, Supplementary Table S4) albeit that the sample size dropped to n=36 with concomitant expected increase in the standard error.

***3. %GLA as a proxy for other fatty acid changes***

Since the proximal omega-6 pathway fatty acid percentage compositions of gamma-linolenic acid (%GLA), linoleic acid (%LA), eicosadienoic acid (%EDA), Dihomo-g-linolenic acid (%DGLA), and arachidonic acid (%AA) are positively correlated, and arachidonic acid is known to be a critical breast milk fatty acid for infant growth and development (Hadley et al., 2016), we re-checked that %GLA was not a correlated proxy for a true latent association with one of these other omega-6 fatty acids. We included all five fatty acids as log(%FA), competitively in the same primary outcome multiple regression model in Bangladesh, and compared the effects and significance. The only significant association of the five fatty acids at p<0.05 was with %GLA (p=0.0039).

**Supplementary Tables**

Supplementary Table S1. Inclusion and exclusion criteria for enrollment eligibility into the Bangladesh PROVIDE cohort.

| **Inclusion criteria** | |
| --- | --- |
| 1 | Mother willing to sign informed consent form |
| 2 | Healthy infant aged 0–7 days old |
| 3 | No obvious congenital abnormalities or birth defects |
| 4 | No abnormal (frequency and consistency) stools since birth |
| 5 | Stable household with no plans to leave the area for the next 1 year |
| **Exclusion criteria** | |
| 1 | Parents are not willing to have child vaccinated at the ICDDR,B field clinic |
| 2 | Parents are not willing to have child’s blood drawn |
| 3 | Parents are planning to enroll child into another interventional clinical study during the period of this trial that could affect the outcomes of this study |
| 4 | Mother not willing to have blood drawn and breast milk extracted |
| 5 | Parents not willing to have field research assistant in home two times per week |
| 6 | History of seizures or other apparent neurologic disorders |
| 7 | Infant received any vaccines before start of study, except BCG |
| 8 | Infant has any sibling currently or previously enrolled in this study, including a twin |

Supplementary Table S2. The 26 fatty acids measured in the breast milk specimens and tested for association with the anthropometric outcomes.**^*^**

| **Fatty Acid Common Name** | **Abbrev** | **Formula** | **Mean %FA ± SD**  **N=683^†^** | **Mean %FA ± SD**  **N=563^†^** | **Inter-assay %CV^‡^** |
| --- | --- | --- | --- | --- | --- |
| **Saturated** |  |  |  |  |  |
| Capric | CAP | C10:0 | 1.16 ± 0.54 | 1.15 ± 0.52 | 26.6 |
| Lauric | LAU | C12:0 | 8.16 ± 3.08 | 8.15 ± 3.01 | 4.7 |
| Myristic | MYR | C14:0 | 8.04 ± 3.13 | 8.01 ± 3.06 | 3.9 |
| Palmitic | PAL | C16:0 | 26.6 ± 3.72 | 26.7 ± 3.72 | 4.6 |
| Stearic | STE | C18:0 | 3.94 ± 0.85 | 3.94 ± 0.83 | 7.2 |
| Arachidic | ARA | C20:0 | 0.14 ± 0.03 | 0.14 ± 0.03 | 4.3 |
| Behenic | BEH | C22:0 | 0.07 ± 0.02 | 0.07 ± 0.02 | 18.1 |
| Lignoceric | LIG | C24:0 | 0.08 ± 0.03 | 0.08 ± 0.03 | 11.1 |
| **ω6-Polyunsaturated** |  |  |  |  |  |
| Linoleic | LA | C18:2ω6 | 11.3 ± 5.03 | 11.3 ± 5.06 | 5.5 |
| γ-Linolenic | GLA | C18:3ω6 | 0.16 ± 0.11 | 0.16 ± 0.11 | 15.5 |
| Eicosadienoic | EDA | C20:2ω6 | 0.41 ± 0.18 | 0.41 ± 0.17 | 10.0 |
| Dihomo-γ-linolenic | DGLA | C20:3ω6 | 0.55 ± 0.17 | 0.55 ± 0.17 | 11.8 |
| Arachidonic | AA | C20:4ω6 | 0.53 ± 0.15 | 0.53 ± 0.14 | 15.4 |
| Docosatetraenoic | DTA | C22:4ω6 | 0.18 ± 0.10 | 0.18 ± 0.10 | 6.4 |
| Docosapentaenoic-n6 | DPA6 | C22:5ω6 | 0.12 ± 0.05 | 0.12 ± 0.05 | 10.7 |
| **ω3-Polyunsaturated** |  |  |  |  |  |
| α-Linolenic | ALA | C18:3ω3 | 0.54 ± 0.40 | 0.54 ± 0.40 | 8.7 |
| Eicosapentaenoic | EPA | C20:5ω3 | 0.06 ± 0.07 | 0.06 ± 0.07 | 25.0 |
| Docosapentaenoic-n3 | DPA | C22:5ω3 | 0.14 ± 0.08 | 0.14 ± 0.08 | 21.5 |
| Docosahexaenoic | DHA | C22:6ω3 | 0.39 ± 0.14 | 0.39 ± 0.13 | 10.9 |
| **Monounsaturated** |  |  |  |  |  |
| Palmitoleic | PLE | C16:1ω7 | 2.91 ± 1.08 | 2.92 ± 1.1 | 4.3 |
| Oleic | OLE | C18:1ω9 | 33.2 ± 4.63 | 33.2 ± 4.63 | 3.1 |
| Eicosenoic | EIC | C20:1ω9 | 0.42 ± 0.16 | 0.41 ± 0.15 | 19.9 |
| Nervonic | NER | C24:1ω9 | 0.15 ± 0.11 | 0.15 ± 0.11 | 47.3 |
| **Trans Fatty Acids** |  |  |  |  |  |
| Palmitelaidic | PLA | C16:1ω7t | 0.06 ± 0.03 | 0.06 ± 0.03 | 82.1 |
| Elaidic | ELA | C18:1t | 0.36 ± 0.26 | 0.37 ± 0.26 | 11.3 |
| Linoelaidic | LLA | C18:2ω6t | 0.32 ± 0.18 | 0.32 ± 0.16 | 7.6 |

**^*^** Each fatty acid was measured as a wt/wt percentage composition of all fatty acids. The values shown are the mean percentage of fatty acid in the milk sample ±SD where the figures are rounded to 2 decimal places for values < 10.0 and 3 sig fig. otherwise.

**^†^** N=683 included all mothers with breast milk samples, N=563 were those whose infants also had primary outcome anthropometry and zinc measures.

**^‡^** The inter-assay % CV (coefficient of variation) was calculated as per Supplementary Methods using published data from OmegaQuant Jackson et al 2016, Int. Breastfeed. J.

Supplementary Table S3. Power to detect an association with each Fatty Acid explaining X% of the variance of the primary outcome via the LASSO variable selection procedure.

|  |  | **% Power to detect Fatty Acid explaining X% of primary outcome variance ΔLAZ (52 – 6 week)** | | | |
| --- | --- | --- | --- | --- | --- |
| **Fatty Acid** | **N simulations** | **X=0.5 %** | **X=1.0 %** | **X=2.0 %** | **X=5.0 %** |
| LA | 1000 | 3.3 | 12.1 | 34.7 | 80.0 |
| GLA | 1000 | 6.9 | 18.3 | 45.5 | 90.6 |
| EDA | 1000 | 5.3 | 15.6 | 41.4 | 90.4 |
| DGLA | 1000 | 7.9 | 22.9 | 53.7 | 96.7 |
| AA | 1000 | 6.5 | 16.5 | 44.4 | 87.7 |
| DTA | 1000 | 3.4 | 11.2 | 32.0 | 81.7 |
| DPA6 | 1000 | 8.2 | 20.7 | 47.6 | 93.1 |
| ALA | 1000 | 4.3 | 13.0 | 37.3 | 80.1 |
| EPA | 1000 | 7.8 | 21.4 | 51.0 | 94.5 |
| DPA | 1000 | 6.4 | 15.8 | 42.8 | 90.6 |
| DHA | 1000 | 6.4 | 19.3 | 47.8 | 91.6 |
| CAP | 1000 | 7.3 | 22.0 | 51.3 | 93.9 |
| LAU | 1000 | 4.6 | 14.0 | 36.5 | 83.3 |
| MYR | 1000 | 7.0 | 16.2 | 40.8 | 88.0 |
| PAL | 1000 | 6.5 | 19.0 | 48.0 | 90.7 |
| STE | 1000 | 6.4 | 19.6 | 47.2 | 90.9 |
| ARA | 1000 | 6.8 | 17.7 | 46.4 | 91.7 |
| BEH | 1000 | 8.1 | 20.7 | 50.5 | 93.8 |
| LIG | 1000 | 6.3 | 16.9 | 47.0 | 93.4 |
| PLE | 1000 | 8.0 | 21.5 | 54.7 | 97.0 |
| OLE | 1000 | 5.9 | 18.4 | 48.4 | 93.2 |
| EIC | 1000 | 5.8 | 16.6 | 44.7 | 88.2 |
| NER | 1000 | 5.4 | 16.5 | 45.6 | 90.2 |
| LLA | 1000 | 6.9 | 19.0 | 45.0 | 89.1 |
| PLA | 1000 | 9.6 | 21.2 | 52.2 | 92.3 |
| ELA | 1000 | 7.1 | 18.9 | 52.7 | 93.1 |
| **Range** |  | **3.3 – 9.6** | **11.2 – 22.9** | **32.0 – 53.7** | **80.0 – 97.0** |

Supplementary Table S4. Comparison of the results for the primary linear growth faltering outcome in the PROVIDE Bangladeshi families for selected Fatty Acid association adjusted for gestational age, and by stage of lactation (corresponding to Figure 3, main text)

| **Stage (model)^*^** | **N** | **Log(%AA)^†^**  **(beta ± se)** | **Log(%DHA)^†^**  **(beta ± se)** | **Log(%GLA)^†^**  **(beta ± se)** |
| --- | --- | --- | --- | --- |
| **Colostrum (minimal)** | **72** | 1.49 ± 0.48  p=0.0031 | -0.70 ± 0.35  p=0.048 | 0.27 ± 0.13  p=0.052 |
| **Colostrum**  **(minimal + gestational age)** | **36** | 0.94 ± 0.81  p=0.26 | 0.11 ± 0.51  p=0.83 | 0.20 ± 0.19  p=0.31 |
| **Transitional (minimal)** | **409** | 0.22 ± 0.20  p=0.27 | 0.07 ± 0.15  p=0.64 | 0.25 ± 0.07  p=0.00036 |
| **Transitional (minimal + gestational age)** | **224** | 0.52 ± 0.28  p=0.063 | -0.15 ± 0.20  p=0.45 | 0.25 ± 0.10  p=0.011 |
| **Mature**  **(minimal)** | **82** | 0.19 ± 0.57  p=0.74 | -0.11 ± 0.41  p=0.79 | 0.48 ± 0.23  p=0.038 |
| **Mature**  **(minimal + gestational age)** | **60** | -0.36 ± 0.68  p=0.60 | 0.08 ± 0.47  p=0.86 | 0.28 ± 0.27  p=0.30 |
| **Total**  **(minimal)** | **563** | 0.40 ± 0.17  p=0.019 | -0.05 ± 0.13  p=0.69 | 0.28 ± 0.06  p=2.7x10^-6^ |
| **Total (minimal + gestational age)** | **318** | 0.42 ± 0.23  p=0.071 | -0.08 ± 0.16  p=0.64 | 0.27 ± 0.08  p=0.00073 |

^*^ Stage: Colostrum < 6 days; Transitional 6-15 days; Mature >15 days. The adjustments in the minimal model are defined in the main text; minimal + gestational age indicates an additional adjustment for gestational age, measured in a subset of infants.

**^†^** AA: Arachidonic Acid; DHA: Docosahexaenoic Acid; GLA: Gamma-linolenic Acid

**Supplementary Figures**

700

693

683

642

594

Did not consent to breast milk study

(-7 )

Insufficient

Sample (-10 )

Loss to follow-up at Week 6 (-41 )

Additional loss to follow-up at Week 52 (-48 )

*Enrolled*

*Breast milk samples collected*

*Breast milk samples assayed*

*Week 6 anthropometry*

*Week 52 anthropometry*

563

*Infant Zinc at week 6 & 18*

Infant Zinc not available at week 6 & 18 (-31 )

PROVIDE - Bangladesh

Supplementary Figure S1. CONSORT Diagram for the study.

Supplemental Figure S2. Plots of the adjusted primary (ΔLAZ 52 – 6 week) and secondary (ΔLAZ 104 – 6 week) outcomes against the percentage of GLA in breast milk (%GLA) in families corresponding to the plots of the unadjusted primary outcome in Figure 2 (a. and b.). The outcome has been adjusted for the ‘Minimal’ set of covariates, see main text. LAZ, Length for age Z score.
